# Impact of 85 kHz versus 125 kHz SHIFT OCTA scan speeds on image quality in retinal diseases and diagnostic reliability of choroidal neovascular membranes

**DOI:** 10.1038/s41598-025-32549-y

**Published:** 2025-12-18

**Authors:** Melanie D. Tran, Nehal Nailesh Mehta, Ines D. Nagel, Mohamed S. Morsy, Amr L. Ali, Anna Heinke, Ruby Munoz, Dirk-Uwe Bartsch, Evan Walker, Lingyun Cheng, William R. Freeman

**Affiliations:** 1 Jacobs Retina Center, Shiley Eye Institute, University of California San Diego, 9415 Campus Point Dr, La Jolla, CA 92093 USA; 2 School of Medicine, University of California San Diego, La Jolla, CA 92037 USA; 3 Viterbi Family Department of Ophthalmology and Shiley Eye Institute, University of California San Diego, San Diego, CA USA; 4 Division of Ophthalmology Informatics and Data Science, Viterbi Family, Department of Ophthalmology and Shiley Eye Institute, University of California San Diego, 9415 Campus Point Drive, La Jolla, CA 92037 USA

**Keywords:** OCTA, SHIFT OCTA, Heidelberg spectralis, Angiotool, Diseases, Health care, Medical research

## Abstract

The purpose of the study was to compare Optical Coherence Tomography Angiography (OCTA) scan speeds of 85 kHz and 125 kHz with respect to image quality, diagnostic reliability, and scan time in patients with retinal diseases. In this prospective cohort study, OCTA images were obtained at both scan speeds in 70 eyes from 40 patients with retinal diseases. Masked expert graders evaluated qualitative parameters including clinical utility, artifacts, and overall image quality. Quantitative parameters including scan time, Heidelberg Q-score, and OCTA-Q score were recorded. In 46 eyes with visible choroidal neovascular membrane in the avascular layer of OCTA, AngioTool (Image J) was used to assess vessel percentage area, vessel junction density, average vessel length, and E-Lacunarity. Acquisition speed of 125 kHz OCTA was significantly faster than that of 85 kHz. There were no statistically significant differences in AngioTool parameters between the two protocols. 125 kHz was significantly better than 85 kHz for image quality with fewer noise artefacts and vessel projection artefacts. In conclusion, 125 kHz SHIFT OCTA offers comparable to better image quality to the 85 kHz OCTA with significantly faster acquisition, potentially improving clinical workflow without compromising diagnostic reliability.

**Supplementary Information:**

The online version contains supplementary material available at 10.1038/s41598-025-32549-y.

## Introduction

Optical coherence tomography angiography (OCTA) has emerged as a valuable non-invasive imaging modality for visualizing retinal vascular networks and pathological neovascularization without the need for dye injection. Previous studies have demonstrated the utility of OCTA in various retinal diseases, including age-related macular degeneration (AMD), retinal vein occlusions, and macular telangiectasia^1–3^.

Recent technological advancements, such as the introduction of the Heidelberg Spectralis SHIFT OCTA (Heidelberg Engineering, Heidelberg, Germany), have enabled OCTA imaging at increased acquisition speeds, including selectable scanning rates of 85 kHz, 125 kHz, and 250 kHz. Prior studies investigating different acquisition speeds of OCTA in healthy eyes have seen decreased acquisition times at higher scan speeds^4,5^. These improvements may enhance patient comfort, decrease motion artifacts, and streamline examination workflow^4^. In addition, slower scan speeds may allow increased visibility of small intraretinal structures.

However, it remains unclear whether increased imaging speeds negatively affect the diagnostic quality in eyes with pathology, especially regarding the clear visualization of choroidal neovascular membranes (CNV). A previous study found that CNV can be visualized at 400 kHz scan speeds^1^. This study aims to compare OCTA imaging at 85 kHz and 125 kHz scan speeds to evaluate whether faster acquisition compromises the quality and diagnostic reliability in patients with retinal diseases including AMD, mild non-proliferative diabetic retinopathy (NPDR), or myopic CNV.

## Methods

### Study design and participants

This prospective study was conducted at the Jacobs Retina Center and Shiley Eye Institute at University of California, San Diego. Patients were eligible for inclusion if they were able and willing to undergo retinal imaging. Patients presenting to retina clinic between March 2025 to May 2025 with AMD, mild NPDR, or myopic CNV were informed of the study. All sequential patients presenting to the retina clinic during the study period were approached using the same standardized communication methods. Diagnosis was confirmed on clinical examination and retinal imaging by a trained retina specialist (WRF). Patients who were willing to participate in the study were given a written informed consent for the study. Institutional Review Board approval from UCSD was obtained (IRB #120516). The study adhered to the tenets of the Declaration of Helsinki for research involving human subjects and complies with Health Insurance Portability and Accountability Act (HIPAA) regulations. 70 eyes from 40 patients were included in the study. The diagnosis of AMD, NPDR, and myopic CNV were chosen since these were the most common presenting cases that were likely to have any changes in OCTA. Patients were excluded if they had significant media opacities or head tremors that impaired the acquisition of high-quality OCTA images. Patients unable to cooperate or follow instructions for stable image capture were also excluded.

### Imaging protocol

**Fig. 1:**
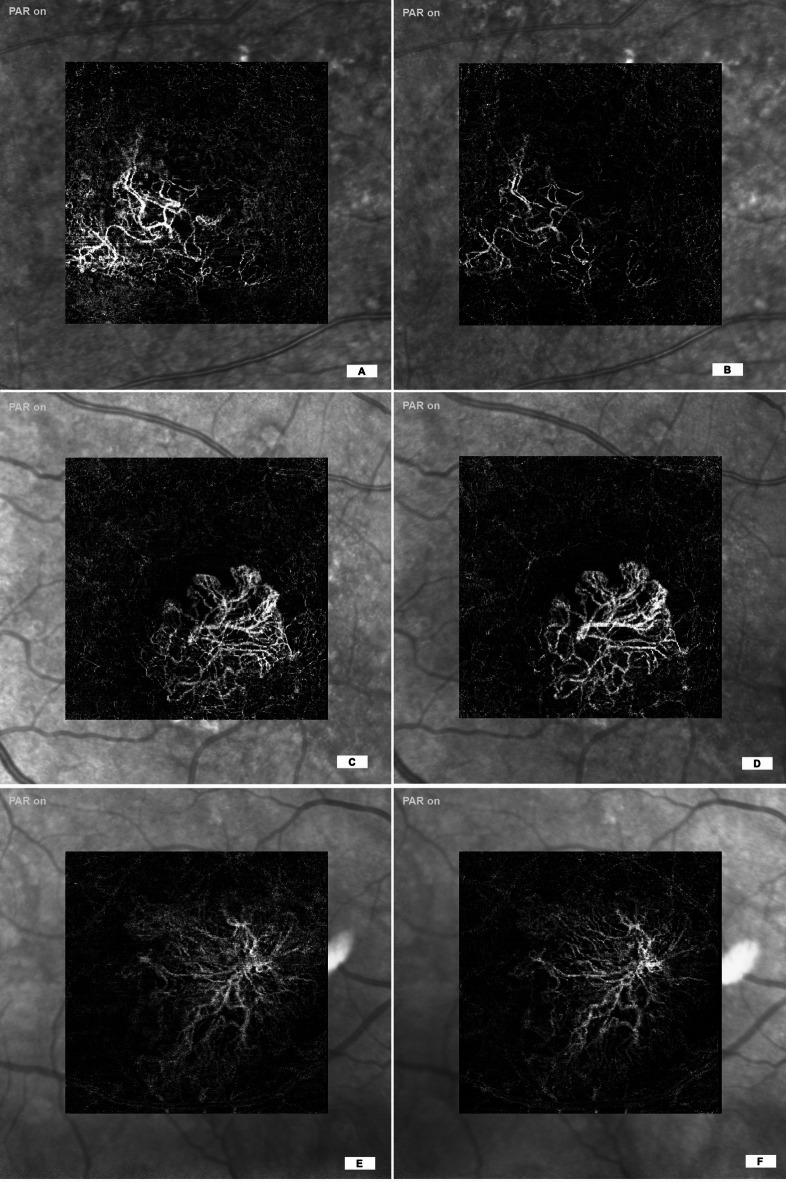
Side-by-side examples of OCTA images obtained at 85 kHz (A,C,E) and 125 kHz (B,D,F) SHIFT scan speeds. These paired images illustrate differences in image quality and visualization of retinal microvasculature between the two acquisition modes.

Each patient underwent OCTA imaging using a Heidelberg Spectralis system (Heidelberg Engineering, Germany) at two different scanning speeds: 85 kHz and 125 kHz SHIFT system. Images were acquired at the same visit to minimize physiological variability. OCTA images were obtained under standardized conditions. Images were acquired by three trained imagers using a standardized protocol. Participants were randomized in a 1:1 ratio, with half imaged first at 85 kHz and the other half at 125 kHz. Each participant underwent sequential imaging without dilation at both scan speeds by the same imager to minimize inter-operator variability. All images were successfully obtained on the first attempt, and no deletions or reacquisitions were required. Each OCTA image was acquired as 10° x 10° volume scan at high resolution mode for 512 × 512 pixel images at mean ART of 5. Each scan was centered at the fovea with a central fixation light set in the machine. It was ensured that the signal quality (Heidelberg Q score) for each scan was more than 25. The segmentation accuracy of each OCTA volume scan was reviewed by a trained retina specialist (NNM) using Heyex, Heidelberg’s proprietary image analysis software (Heidelberg Engineering, Germany, Version 2.6.6, www.HeidelbergEngineering.com). Eight eyes required manual correction of Bruch’s Membrane segmentation in both 85 kHz and 125 kHz SHIFT OCTAs. The circle tool of Heyex was used to modify the segmentation across two scans within the region of error, after which the entire OCTA volume was rechecked. As demonstrated previously, the Heyex software can propagate segmentation corrections across the entire volume once two scans are manually corrected^6^. Only two eyes required additional manual correction for accurate segmentation, which was achieved by manually resegmenting all scans within the affected region of the volume scan. The avascular layer of each scan was then exported in ‘png’ image format using the “transverse analysis” export current view option in Heyex 2 (Fig. 1). All patient information was anonymized and relabeled for AngioTool analysis (AngioTool 2.0, upgraded version by Jack Bendtsen, https://github.com/jbendtsen/AngioTool-Batch) based on the original version developed National Cancer Institute, Center for Cancer Research, Bethesda, USA) and human grading^7^. Scan times were recorded in the log files of the instrument and anonymously analyzed by Heidelberg Engineering.

### Image quality and analysis

A total of 70 paired images (85 kHz and 125 kHz) were independently reviewed by three expert graders (IN, AA, MM), masked to scan speed. Each image pair was evaluated across four domains: overall image quality, clinical utility, vessel projection artifacts, and other imaging artifacts^8^. Qualitative comparison by human graders focused on the visibility of the neovascular membrane, definition of vascular structures, and the presence of potential information loss. Grading was performed using a 3-point ordinal scale (125 kHz superior, equal, 85 kHz superior). For CNV cases, additional qualitative assessment focused on the visibility of the neovascular membrane, clarity of vascular structures, and presence of potential information loss. Agreement between graders was assessed using pairwise percent agreement and Cohen’s κ. In cases of disagreement, no forced consensus was applied; instead, all individual grader responses were retained for statistical analysis. This approach allowed for modeling grader preference while accounting for inter-grader variability.

Quantitative parameters, including Heidelberg Q-score (quality-score) as well as OCTA Q score, were recorded for each image in Heyex-2 and compared accordingly^9^. The Q score is the signal-to-noise ratio of the recorded signal over the noise background level and is measured on a scale. The Q-score is proportional to the logarithm of the maximum signal divided by the maximum background noise^10^. According to the manufacturer’s guidelines, a score of 25 or above is recommended for adequate image reliability^10^. Diagnostic reliability was evaluated in 46 eyes with a visible choroidal neovascular identified in the avascular retinal layer. An expert human grader manually delineated the CNV area on both 85 kHz and 125 kHz OCTA scans to ensure accurate and consistent region-of-interest selection. The delineated areas were then analyzed using AngioTool (ImageJ plugin, National Cancer Institute, USA) to quantify CNV morphology. The following parameters were extracted and compared between scan speeds based on a previously validated protocol: vessel percentage area, vessel junction density, average vessel length, and E-lacunarity^11^. These quantitative vascular metrics served as objective surrogates of diagnostic reliability.

### Statistical analysis

All statistical analyses were performed using JMP statistical software (JMP^®^, Version 18. SAS Institute Inc., Cary, NC) or the R programming language for statistical computation, version 4.4.0 (R Core Team (2024). R: A Language and Environment for Statistical Computing. R Foundation for Statistical Computing, Vienna, Austria.). R packages ‘irr’, ‘lme4’, ‘ordinal’, ‘ggplot2’, and ‘brms’ were utilized as part of the analysis.

Continuous variables were summarized as means with standard errors. Paired t-tests were used to compare quantitative measurements (OCTA Quality, Q Quality, scan acquisition time, vessel percentage area, vessel junction density, average vessel length, and E-Lacunarity) between the 85 and 125 kHz scan protocols.

For ordinal image quality assessments by graders, results were coded into three categories: 85 better, 125 better, or equal. A p-value less than 0.05 was considered statistically significant. Inter-grader agreement was assessed using Cohen’s Kappa for comparisons between two graders. An analysis was employed to assess grader preference between 85 kHz and 125 kHz, after excluding “Equal” grader responses. Generalized linear mixed-effects models were used to evaluate the probability of graders selecting “125 kHz” versus “85 kHz”. The model included a fixed effect for prompt to assess inter-prompt image preference. Random intercepts for patient and grader were incorporated to account for repeated measures and inter-grader variability. Image preference probabilities were derived from model-estimated log-odds.

## Results

### Demographic data

A total of 40 patients were included in the study, comprising of 15 males (37.5%) and 25 females (62.5%). The mean age was 76.8 ± 10.1 years. 31 patients (77.5%) identified as White, 5 (12.5%) as Asian, 2 (5.0%) as Other, and 2 (5.0%) did not report their race. A total of 70 eyes were included in the study. Diagnoses included AMD in 59 eyes, myopic CNV in 2 eyes, and mild NPDR in 9 eyes. A summary of the ocular history of the included patients, including the presence of dry eye disease, cataracts, vitreous floaters, syneresis, asteroid hyalosis, and history of prior ocular surgery, is provided in Supplemental Table S1.

### Quantitative analysis

The 85 kHz OCTA protocol had a mean acquisition time of 67.15 ± 28.22 s, while the 125 kHz protocol had a mean acquisition time of 46.02 ± 16.33 s, representing a statistically significant reduction in acquisition time (*P* < 0.0001). The 125 kHz protocol was approximately 31.5% faster than the 85 kHz protocol (Table 1).

**Table 1:** Comparison of OCTA scan speed metrics between the 85 and 125 scan protocols.

Metric	85 Protocol (Mean ± SD)	125 Protocol ( Mean ± SD)	Percentage improvement in speed	*p*-value
OCTA scan speed (seconds)	67.15 ± 28.22	46.02 ± 16.33	31.5	< 0.0001

The 85 kHz OCTA protocol demonstrated significantly higher mean scores for both OCTA Quality (33.66 vs. 31.56; mean difference = -2.10, SE = 0.21, *p* < 0.0001) and Q Quality (33.89 vs. 31.86; mean difference = -2.03, SE = 0.39, *p* < 0.0001) compared to the 125 SHIFT protocol. However, there were no statistically significant differences between AngioTool parameters of vessel percentage area (37.29 vs. 34.37; mean difference = -2.92, SE = 1.69, *p* = 0.091), vessel junction density (0.00063 vs. 0.00059; mean difference = -0.000033, SE = 0.000033, *p* = 0.33), average vessel length (246.07 vs. 201.71; mean difference = -44.37, SE = 37.09, *p* = 0.2379), or E-Lacunarity (0.390 vs. 0.429; mean difference = 0.039, SE = 0.036, *p* = 0.294) (Table 2).

**Table 2:** Comparison of AngioTool parameters between the 85 and 125 kHz scan protocols.

AngioTool Metric	85 Protocol (Mean)	125 Protocol (Mean)	Mean Difference (SE)	*p*-value
Vessel % Area	37.29	34.37	-2.92 (1.69)	0.091
Vessel Junction Density	0.00063	0.00059	-0.000033 (0.000033)	0.33
Average Vessel Length	246.07	201.71	-44.37 (37.09)	0.238
E-Lacunarity	0.39	0.429	0.039 (0.036)	0.294

### Qualitative assessment

For image quality, the inter-grader pairwise percent agreement was moderate at 58.6% across all rater pairs, with Cohen’s Kappa indicating slight to fair agreement beyond chance.

**Fig. 2:**
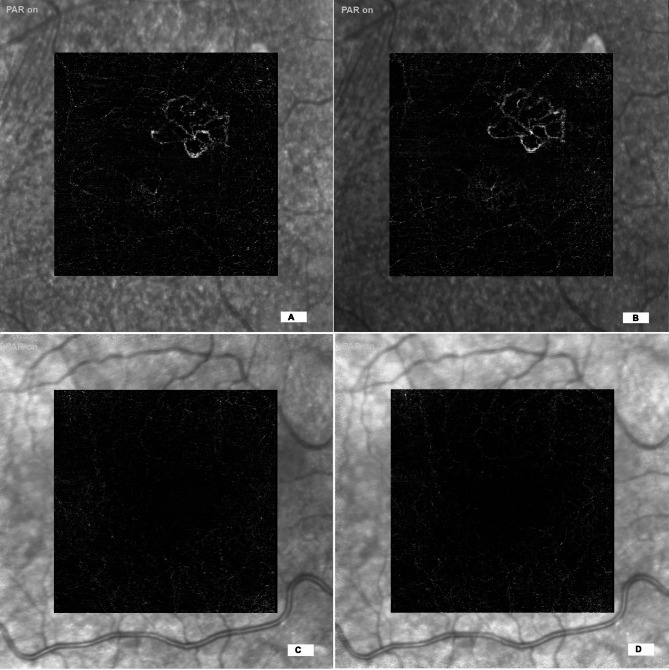
Sample avascular layer OCTA images exported using transverse analysis in Heyex 2 software. (A,B) Show images acquired at 85 kHz and 125 kHz, respectively, from a case with visible choroidal neovascularization (CNV). Panels C and D show images acquired at 85 kHz and 125 kHz, respectively, from a case without CNV.

**Fig. 3:**
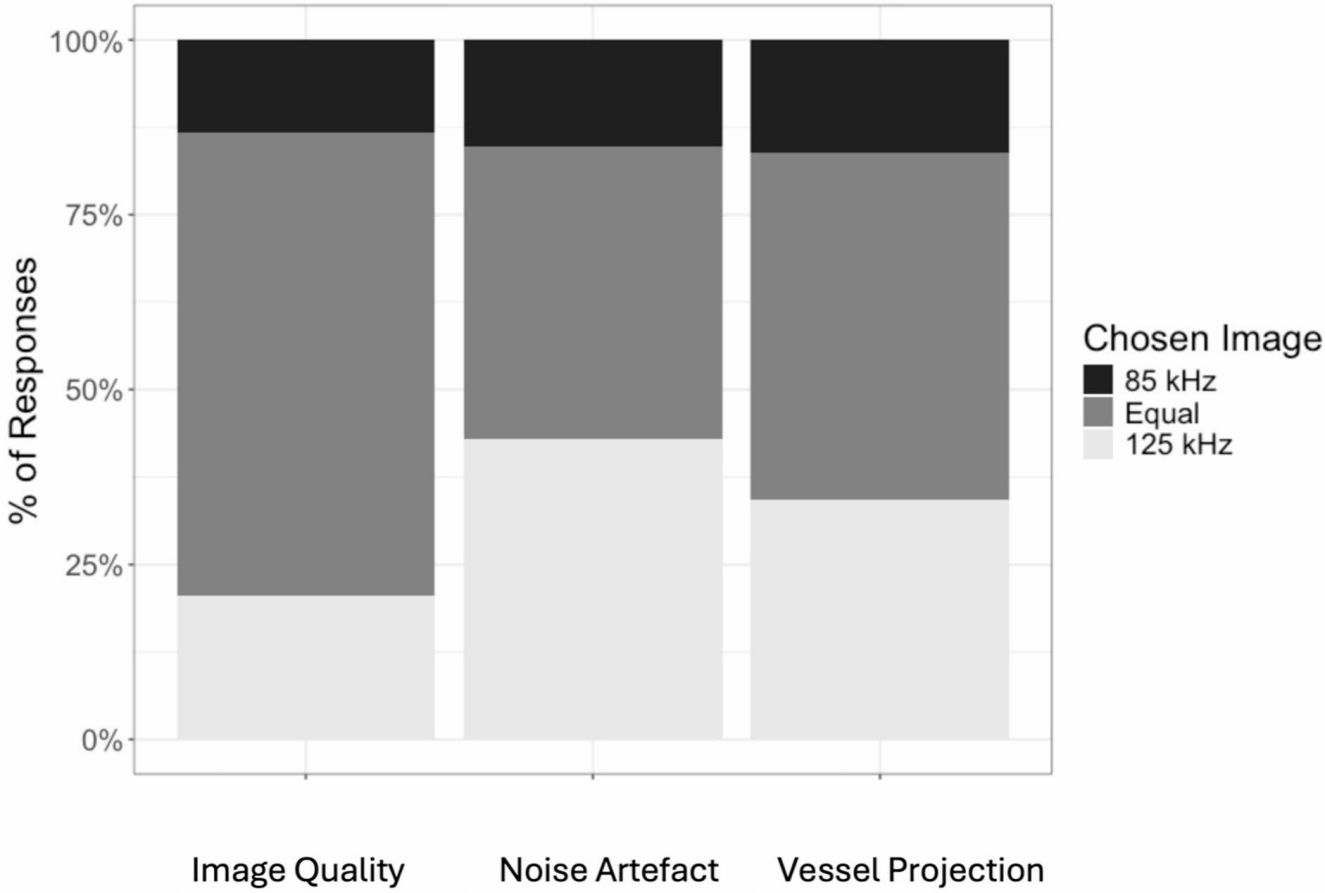
The bar chart shows the percentage of paired image assessments that were rated as “85 protocol better,” “125 protocol better,” or “equal quality” by human graders, for the respective categories of image quality, noise artefact and vessel projection artifacts.

**Fig. 4:**
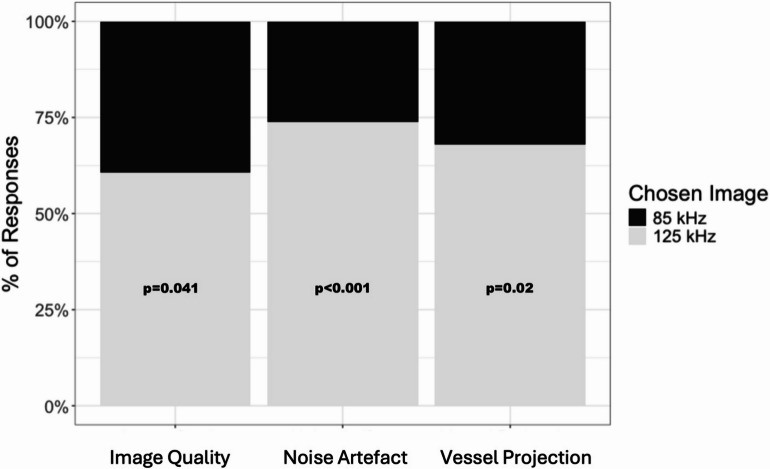
The bar chart shows the percentage of paired image assessments that were rated as “85 protocol better,” or “125 protocol better” by human graders, for the respective categories of image quality, noise artefact and vessel projection artifacts. All three qualitative parameters were statistically significant, with higher odds of graders preferring the 125 kHz protocol.

To assess grader preference between the 85 kHz and 125 kHz OCTA protocols, responses were analyzed across three qualitative domains: image quality, noise artifact, and vessel projection, excluding cases in which graders rated the two protocols as “Equal.” Representative OCTA images acquired at 85 kHz and 125 kHz scan speeds are shown in Fig. 2. The analysis reflects results from the model excluding an intercept, where grader preference was inferred based on the directionality of responses favoring one protocol over the other. All three qualitative parameters were statistically significant, with higher odds of graders preferring the 125 kHz protocol: image quality (Estimate = 0.776, *p* = 0.041), noise artifact (Estimate = 1.408, *p* < 0.001), and vessel projection (Estimate = 1.080, *p* = 0.002). These findings indicate a consistent and significant preference for the 125 kHz protocol when neutral responses were excluded. Figures 3 and 4 demonstrate that, in both the presence and absence of directional choices, graders consistently favored the 125 kHz protocol over the 85 kHz protocol.

## Discussion

In this study, we compared two OCTA scan protocols (85 and 125 kHz) across multiple quantitative and qualitative image quality metrics. SHIFT is a newer imaging modality available only on recently manufactured Heidelberg Spectralis systems and represents the latest federally approved advancement for OCTA acquisition on this platform. In July 2024, the U.S. Food and Drug Administration (FDA) granted clearance for the OCTA Module with SHIFT technology using a preset scan speed of 125 kHz. However, as a recent innovation, SHIFT is currently available only at select centers equipped with the latest generation Spectralis devices, limiting widespread access and published data across diverse patient populations. Our study is therefore among the first to systematically evaluate the performance of the 125 kHz SHIFT OCTA setting, specifically testing the manufacturer’s claim that it enables a substantial reduction in acquisition time without compromising image quality.

As per the manufacturer, the 125 kHz OCTA scan speed offers up to 30% faster image acquisition compared to the 85 kHz scan speed^12^. Our study had similar results with 31.5% faster acquisition rates on SHIFT 125 kHz than 85 kHz protocol. Since acquisition times were directly obtained from the device’s internal log files and anonymized for analysis by Heidelberg Engineering, potential human measurement errors were eliminated. Variability in scan acquisition times within the same protocol (e.g., 46.01 ± 16.33 s for the 125 kHz protocol) was primarily attributable to patient-related factors such as fixation instability, blinking, fatigue, or repositioning for optimal imaging. Additionally, longer scans were more difficult for some patients to tolerate, which may have increased the likelihood of interruptions. These influences were present across both the 85 kHz and 125 kHz protocols, but their impact was likely less pronounced in the faster 125 kHz protocol given its shorter baseline acquisition time.

Both OCTA Quality and OCT Quality scores were significantly higher for the 85 kHz protocol, indicating better signal-to-noise ratio (SNR). The higher SNR observed with slower 85 kHz scans was likely due to longer integration times per A-scan, which allow for greater signal averaging and reduce the relative contribution of random noise^13^. In OCTA, slower scan speeds, or lower A-scan rates, result in higher Q scores, which are quantitative measures of image SNR and quality. Consistent with this, the 85 kHz protocol demonstrated higher Q scores than the faster 125 kHz protocol. This was consistent with a previous study similarly evaluating acquisition speeds at 85 kHz and 125 kHz in 201 healthy eyes, in which mean Q scores were significantly lower at faster speeds^4,5^. Importantly, all images included in our analysis had Q scores greater than 25, which is generally considered the clinical cutoff for good-quality images^10^. The average Q scores for the 85 kHz and 125 kHz protocols were 33.89 and 31.86, respectively. Although a difference in Q scores was observed between the two scan speeds, both produced images of sufficient quality for clinical use. It should be noted that the Q score does not represent a consensus parameter for image quality. It is merely a measure of raw signal-to-noise strength and does not account for focus errors, suboptimal optical alignment, or erroneous layer segmentation in imaging^4,14^. A previous study found that Q scores may be influenced by factors such as blink artifacts, motion, segmentation errors, and poor microvasculature visibility, rendering them insufficient for as a metric of image quality in certain cases despite high numerical scores^14^. Factors such as increased susceptibility to motion artifacts and reduced patient tolerance of longer scans may mitigate the theoretical advantage of higher Q scores at slower acquisition speeds, underscoring the balance between acquisition speed, quantitative image quality, and clinical applicability in OCTA imaging. Overall, while these findings may suggest that slower acquisition speeds may yield higher-quality images as measured by the Q score, additional quality metrics or manual review, such as human grading, may be necessary to fully assess image reliability.

When assessing vascular metrics such as vessel percentage area, vessel junction density, average vessel length, and E-Lacunarity, there were no statistically significant differences between the two protocols, suggesting that these vascular metrics are relatively robust to the scan protocol used. The correlation coefficients for these parameters ranged from moderate to high (e.g., vessel percentage area, *r* = 0.65; E-Lacunarity, *r* = 0.88), suggesting that the two scan protocols generally provide consistent information for the vascular features.

The results of human grading were consistent with a previous study in which human graders also rated images of healthy eyes to be of equal or better quality (92.5%) at the 125 kHz rate in comparison to the 85 kHz rate^4^. The variability in inter-rater agreement observed across assessments may be attributed to several factors. The inherently subjective nature of qualitative OCTA interpretation likely contributed to inconsistencies. Differences in how graders weighed features such as contrast, noise, and artifact severity may have led to divergent assessments, especially in borderline cases. When excluding neutral responses, a greater proportion of grader preferences favored the 125 kHz protocol over the 85 kHz, suggesting enhanced perceived image clarity, reduced noise, and improved visualization of microvascular structures at the higher scan rate. Interestingly, a previous study comparing the 20 kHz and 85 kHz OCT scan rates found that the 20 kHz protocol yielded improved grading results^15^.

These findings highlight not only subjective improvements in image quality with faster acquisition but also raise important considerations for clinical practice. Beyond, faster 125 kHz acquisition speeds may enhance clinical efficiency and workflow while improving patient comfort through shorter imaging times. This is particularly beneficial for older patients or those with limited fixation or poor cooperation, as it helps reduce motion artifacts. Additionally, higher acquisition speeds may increase sensitivity to detecting elevated flow velocities in the choroid and choriocapillaris^16^. Another advantage of higher scan speed is greater tolerance for imaging in patients with retinal disease. Patients with retinal diseases like AMD may have dry eye disease as well^17^. Longer scan times can increase the period that patients need to keep eyes open and reduced blink rate for the duration of scan, contributing to discomfort and movement during scan acquisition and chances of lower scan quality^18^.

A limitation of this study is the restriction to patients who consented to participate, which limited the sample size and may introduce selection bias. The study aimed to minimize selection bias by approaching all eligible patients in a sequential manner using consistent communication protocols. However, some degree of selection bias may have been introduced due to the exclusion of patients with media opacities, significant tremors, or poor cooperation, as these factors impaired the ability to obtain adequate OCTA images. These exclusions were necessary to ensure image quality for accurate analysis, but they may limit the generalizability of the findings to broader patient populations, particularly those with more advanced disease or comorbidities that affect image acquisition. Consequently, a more robust analysis with greater statistical power could be achieved by including a larger and more diverse sample size in future studies. Additionally, this study was conducted at a single center, which may limit the generalizability of the findings to other clinical settings or institutions.

In conclusion, the 125 kHz scan protocol offers significantly faster image acquisition compared to the 85 kHz protocol while maintaining equal or superior image quality, with fewer noise and vessel projection artifacts, supporting its use as a more efficient and reliable option in clinical and research settings. Notably, while the 85 kHz protocol demonstrated higher signal-to-noise ratios, subjective assessments of image quality consistently favored the 125 kHz protocol. Future studies may include investigating the comparative performance of OCTA imaging at 125 kHz and higher scan speeds, such as 250 kHz, to further evaluate the trade-offs between imaging speed and diagnostic accuracy.

## Supplementary Information

Below is the link to the electronic supplementary material.

Supplementary Material 1

## Data Availability

All relevant data is listed in the manuscript. Additional inquiries can be directed towards the corresponding author.

## Abbreviations

OCTA: Optical coherence tomography angiography
AMD: Age related macular degeneration
CNV: Choroidal neovascular membranes
NPDR: Mild non proliferative diabetic retinopathy
HIPAA: Health Insurance Portability and Accountability Act
FDA: U.S. Food and Drug Administration
SNR: Signal-to-noise ratio
