# Supplementary material for: Impact of 85 kHz versus 125 kHz SHIFT OCTA scan speeds on image quality in retinal diseases and diagnostic reliability of choroidal neovascular membranes
Source: Sci Rep. 2025 Dec 18;16:2735. doi: 10.1038/s41598-025-32549-y (PMC12823581; doi:10.1038/s41598-025-32549-y)
Supplement: Supplementary file 1 — Supplementary Material 1 [file 41598_2025_32549_MOESM1_ESM.pdf]

Supplementary Table S1. Summary of ocular history of included eyes.

**Ocular History**	**Number of Eyes**
**Surgery**	
Cataract surgery	42
Vitrectomy	9
Trabeculectomy	1
**Lens Status**	
Pseudophakic	43
Cataract present	27
**Other Ocular History**	
Dry eye disease	39
Vitreous floaters	19
Vitreous syneresis	22
Asteroid hyalosis	0
